# Ambivalent User Needs as a Challenge and Chance for the Design of a Web-Based Intervention for Gaming Disorder: Qualitative Interview Study With Adolescents and Young Adults

**DOI:** 10.2196/63258

**Published:** 2025-05-26

**Authors:** Birte Linny Geisler, Kay Uwe Petersen, Sara Hanke, Simon Schurer, Anne Schreiber, Christine Lämmle, Anil Batra, Tobias Renner, Isabel Brandhorst

**Affiliations:** 1 Department of Child and Adolescent Psychiatry, Psychosomatics and Psychotherapy University Hospital Tübingen Tübingen Germany; 2 German Center for Mental Health (DZPG) Partner Site Tübingen Tübingen Germany; 3 Department of Psychiatry and Psychotherapy Section of Addiction Medicine and Addiction Research University Hospital Tübingen Tübingen Germany

**Keywords:** gaming disorder, user-centered design, self-guided web-based intervention, adolescents, young adults, youth, treatment needs, digital intervention, artificial intelligence, AI

## Abstract

**Background:**

In Germany, there are still many young people with gaming disorder (GD) who do not use or cannot access existing treatment services. Given the increasing prevalence of internet use disorders and GD, especially among young people in Germany, there is a need to provide additional low-threshold treatment options that are easily accessible anywhere. Web-based interventions (WBIs) can be used to achieve this goal.

**Objective:**

The aim of this study was to explore the treatment needs of young people with GD in Germany and derive implications for the development of a self-guided WBI for GD.

**Methods:**

Using a qualitative study design, we conducted a focus group with 3 young male adults and semistructured individual interviews with 3 male adolescents. Data were analyzed using qualitative content analysis. The reporting of this study followed the COREQ (Consolidated Criteria for Reporting Qualitative Research) guidelines.

**Results:**

Participants’ expectations of web-based help in general and of a self-guided WBI for GD revealed a wide variety of sometimes conflicting user needs. For example, by analyzing participants’ experiences with successful strategies, we found that external stabilizers (eg, parental control and support group meetings) were helpful in managing GD. However, with regard to a WBI, participants described it as a barrier if the WBI created “too much pressure.” On the other hand, “not enough pressure” (ie, not enough external control) was also mentioned as a barrier. The belief that gaming is rewarding and that only equally rewarding activities are successful alternatives to gaming is in tension with the fact that changing problematic gaming behavior can be stressful and not feel rewarding at all. The data also showed that, on the one hand, a WBI should be designed to be attractive (eg, by incorporating gaming elements) but that it should not be too attractive as this, in turn, could trigger GD.

**Conclusions:**

A self-guided WBI for GD should consider and address conflicting user needs. Ambivalence of needs in the face of coping with GD should not be seen as a problem but as a normal part of a change process and, therefore, actively integrated into the WBI concept and storyline.

**Trial Registration:**

German Clinical Trials Register DRKS00032334; https://drks.de/search/en/trial/DRKS00032334

## Introduction

### Background

According to the *International Classification of Diseases, 11th Revision* (code 6C51), gaming disorder (GD) can be defined as an online or offline gaming behavior that involves reduced control over gaming, an increase in the prioritization of gaming over other life interests and activities, and the continuation or escalation of gaming despite its negative consequences. Gaming behavior significantly interferes with important areas of life, such as family, education, and work. To be diagnosed with GD, the behavior must typically occur over a period of at least 12 months [1]. In addition to GD, there are other problematic behaviors that can occur in the context of internet use, including those related to social media, online shopping, online pornography, and online gambling. The term internet use disorder (IUD) is sometimes used as an umbrella term for these problem areas. In some cases, IUD is also used as a separate disorder category because users often problematically use multiple internet applications at the same time. The relevance and prevalence of IUD and GD is increasing, especially since the COVID-19 pandemic [2-4].

In Germany, it can be assumed that young people with IUD or GD are still not adequately cared for due to the lack of specialized care centers [5]. During the COVID-19 pandemic, the prevalence of GD increased rapidly among young people [6]. In 2023, there was an estimated 4.3% of individuals aged 10 to 17 years in Germany who had GD. A total of 11.1% met the *International Classification of Diseases, 11th Revision*, criteria for risky gaming behavior, which corresponds to approximately 680,000 affected children and adolescents [7]. Therefore, there is a need to provide low-threshold treatment options that are easily accessible anywhere. Web-based treatment options are ideal for achieving this goal.

With the Online Bridge project (in German: Onlinebrücke), the authors of this paper created Breaking the Game, an innovative treatment offer in the form of a self-guided web-based intervention (WBI; Textbox 1). WBIs can be defined as digital programs on the internet aimed at treating or counseling people with (mental) health problems [8]. Breaking the Game focuses on adolescents and young adults aged ≥12 years with symptoms of GD. The first part of the project, focusing on the development of the WBI, ran from November 2021 to December 2023 and included the qualitative study presented in this paper. This study was registered in the German Clinical Trials Register (DRKS00032334). The project was funded by the Ministry of Social Affairs, Health, and Integration of the state of Baden-Württemberg in Germany.

**Types of web-based interventions (WBIs).**

- Guided WBIs include human therapists providing treatment via webcam, telephone, email, or messages [9,10], such as for internet use disorder (IUD) treatment in the form of a manualized online counseling service using motivational interviewing techniques [2,11,12].
- Self-guided WBIs do not involve a human therapist but provide a predesigned program [9,10], such as an app-based intervention for IUD treatment [2,13].
- Blended WBIs combine digital and analogue treatment or self-guided and guided elements [9,10], such as for IUD treatment as a stepped-care approach combining an app-based self-guided program with webcam counseling [2,14].

However, in conceptualizing the WBI, the research group was faced with questions that could not be answered by the research literature at the time. In WBIs for GD, treatment is offered over the internet, where gaming itself takes place. This distinguishes GD from other (mental) health problems addressed in WBIs, such as diabetes or depression. From the experience of the German WBI OMPRIS, which offered webcam-based counseling, we know that guided WBIs for IUD and GD can work successfully. However, due to the potentially noncommittal nature of the internet environment, aspects of motivation should be considered when developing a successful web-based treatment program [11]. The question was how this could be applied to a *self-guided* WBI for GD. In a self-guided WBI, there is no contact with a counselor or therapist, which could increase the risk of leaving the WBI with a single click. In addition, the question was raised of how to design an attractive WBI for treating GD so that young people affected by GD who are familiar with well-designed games would find it attractive enough to return to it regularly.

In addition, a high variability of characteristics within the target population of young people with GD had to be considered, including age, personality traits, comorbidities, educational background, mental health literacy, and gender [15-17]. Research shows that the development of GD is influenced not only by the amount of time spent online [4] but also by the interaction of several risk and influencing factors [18]. Biographical influences, such as family factors, play a role [19]. For example, the parent-child relationship is an important factor in both the development and maintenance of IUDs [20]. Gender differences in the development and maintenance of GD should be considered, such as the level of impulsivity and impaired inhibitory control or the role of aggressive feelings and craving [21]. In addition, many people do not yet play games pathologically but already do so in an uncontrolled manner [22]. Problem gamers should also be reached with help offers to prevent them from developing GD.

Research into the treatment of GD has examined not only analogue but also digital treatment options. Studies show that both adults and young people with mental health problems can be effectively reached through WBIs, possibly even more effectively than through analogue interventions. For example, an Australian study of adolescents’ attitudes toward online therapy found that 72% of the adolescents surveyed would use online therapy if they had mental health problems. A third would even prefer online therapy to traditional face-to-face options, citing reduced stigma and increased accessibility [23]. Adolescents and young adults already spend a significant amount of time online. When seeking help, it is plausible that young people will (as a first step) turn to the internet as a familiar environment rather than to a local therapist. Therefore, WBIs can fill a gap in care, especially when it comes to reaching young people with mental health problems [24,25].

Studies suggest that WBIs for IUD and GD may also be helpful and effective [2,26], for example, in preventing GD by improving self-regulation [27]. As people with IUD or GD spend even more time online than average, it is likely that treatment services offered online are accessible at a lower threshold than analogue treatment services [11]. In addition, IUD and GD are associated with comorbidities such as depression, attention-deficit/hyperactivity disorder, and social phobia [28]. It is reasonable to assume that people with IUD or GD combined with these comorbidities may have additional problems seeking analogue therapy. Therefore, easily accessible WBIs may be an important treatment option.

The development of a self-guided WBI differs in many ways from the development of a guided WBI (eg, webcam-based individual counseling; Textbox 1) [8]. Self-guided WBI content must be predesigned and standardized, with the risk that the final version will not meet all the needs of a heterogeneous target group. This may be the reason poor adherence to self-guided WBIs remains a problem [29]. However, self-guided WBIs have a number of advantages that distinguish them from other intervention types. They are cost-effective because they do not require human therapists. They can be used at the user’s own pace and needs, with time flexibility and little effort and without long waiting times [9,30]. Users of self-guided WBIs can manage their treatment processes independently, which can increase users' autonomy, self-determination, and self-efficacy. In addition, self-guided WBIs can be used anonymously and without commitment, which is likely to increase willingness to register. Studies suggest that guided WBIs involving human therapists are more effective than self-guided WBIs (ie, they achieve higher rates of adherence) [9,10,31-35]. However, other research shows that the design of the intervention determines its ultimate effectiveness [9,36]. In the area of disordered gambling, a study showed that intensively designed self-guided WBIs with more than 6 thematic modules were more effective than short and less intensively designed face-to-face options [37]. This illustrates the importance of specific design choices for the subsequent effectiveness of a WBI. Therefore, although research suggests that WBIs are generally effective, this proved to be insufficient information for the development of a new self-guided WBI for a young German target group because of the lack of detailed information from the users’ perspective about the assumed effectiveness based on the WBI design.

Participatory and collaborative approaches are playing an increasingly important role in the development and research of treatment options in psychiatry and psychotherapy [38-40]. However, the involvement of future users in the development of treatment services for GD and IUD from the outset has long been lacking in German studies. This applies to both analogue and digital services. To our knowledge, no study has yet investigated the treatment needs of young Germans with GD symptoms regarding a self-guided WBI using a participatory approach before and during the WBI development process. In general, participatory approaches (eg, by using qualitative methods that invite people with IUDs to share their personal experiences with IUDs and their treatment needs) are still rarely used in German web-based IUD treatment research. Dreier and Wölfling [2] conducted a qualitative study on patients’ perceptions of online therapy. However, this analysis showed how patients perceived treatment services after they had already been developed. Chen et al [41], who developed a self-guided WBI for adolescents with GD, involved end users by observing participants using the first version of the WBI and conducting a usability survey. Hanke et al [42] conducted a survey to investigate the needs of parents of adolescents with IUD regarding a WBI. However, there are still WBIs for GD that have not been developed based on a user-centered or human-centered design approach [43,44], which involves the cocreation of an intervention with end-user representatives [45,46]. Therefore, to develop a self-guided WBI for young Germans with GD, the research group lacked information that can only be provided by people with GD and potential users themselves.

Due to the presented research gaps, especially regarding the young German target population with GD, a qualitative interview study with German adolescents and young adults with GD symptoms was conducted within the Onlinebrücke project to collect information from the perspective of potential users to be included in the development of the self-guided WBI Breaking the Game. The interim and final results of the qualitative study were incorporated into the parallel development of the WBI. In addition to integrating the results of this study, we developed the WBI in close collaboration with addiction counseling centers.

### Study Objectives

The aim of this qualitative study was to understand the treatment needs of German adolescents and young adults affected by GD to inform the development of a self-guided WBI for a German target group. The overall research question was as follows: What user needs must be considered when developing a target group–oriented self-guided WBI for adolescents and young adults with GD symptoms in Germany?

This led to the following subquestions: (1) What are the treatment needs of adolescents and young adults with GD symptoms in Germany regarding a self-guided WBI for GD? (2) What are the implications for the development and design of a self-guided WBI for GD for a young German target group?

## Methods

### Study Design

A qualitative interview study was conducted. One focus group and 3 semistructured interviews were used for data collection as these methods are suitable for capturing people’s perspectives and experiences on a predefined topic. Qualitative content analysis according to Kuckartz and Rädiker [47] was used to analyze the data as this method allows for deductive and inductive development of coding categories. In this way, it was possible to find unexpected results as well as follow predefined categories based on the research questions. The ability to analyze the data in a more interpretive way when needed helped gain insights into user needs that are often hidden in implicit statements [47]. The COREQ (Consolidated Criteria for Reporting Qualitative Research) guidelines were followed in reporting this study (Multimedia Appendix 1) [48].

### Ethical Considerations

Approval was granted by the ethics committee at the Medical Faculty of the Eberhard Karls University of Tübingen and University Hospital Tübingen on February 15, 2023, under the reference number 843/2022BO2. This study was conducted in accordance with the Declaration of Helsinki. Participants gave their informed consent to participate before data collection. In the case of minors, the parents also signed the consent form. Participants received no monetary compensation for taking part in this study. The interviews were transcribed pseudonymously by a certified transcription service (Amanu). The transcripts were stored on protected servers at University Hospital Tübingen.

### Study Population

The aim was to interview adolescents and young adults with GD symptoms as this was the target group for the Breaking the Game WBI. The young adults in the study population were recruited through a self-help network in Germany by author IB as the research group leader. Adolescents were recruited at a German child and adolescent psychiatric outpatient clinic by author SS, who was a master’s student in psychology. Finding interviewees proved to be a challenge. Due to limited time and personnel resources, the search focused on institutions within the health care system and did not extend to settings of everyday life (eg, schools, universities, and youth centers). Finally, participants were selected through a combination of purposive sampling (ie, directly contacting an IUD support group where people who met the inclusion criteria could be found) and convenience sampling (ie, spreading the word among clinic staff to look for young patients with GD symptoms). During recruitment, the inclusion criteria were adjusted to include current or past experience with GD symptoms and an age range that approximated the age range of future WBI users. For example, although the former age range of the WBI was 12 to 21 years (later changed to ≥12 years), participants aged >21 years were recruited because of their experience with GD at a younger age and their interest in speaking with researchers. A diagnosis of GD was not required. One potential participant dropped out of the study. He did not attend the agreed appointment, and no alternative date could be arranged. The time frame for data collection was 6 months. Data collection was completed when 3 male young adults and 3 male adolescents had been interviewed. The heterogeneity of the study population in terms of GD experience was acceptable. However, it can be assumed that, if recruitment had also taken place outside the health care system, female participants would most likely have been reached. In Germany, there are still more men with GD than women with GD in the health care system.

The sample in this study consisted of 3 male young adults in their 20s (P1, P2, and P3) and 3 male adolescents between the ages of 12 and 16 years (P4, P5, and P6). Textbox 2 provides more details.

**Sample description.**

- P1 (male; in his 20s; university student) first noticed symptoms of gaming disorder (GD) at the beginning of his studies. During exam periods, he used games to procrastinate. During this time, he felt the need to change. Therefore, several years ago, he got rid of his notebook. Since then, his gaming problems have gradually improved. However, in retrospect, there were already symptoms of GD during his school years.
- P2 (male; in his 20s; university student) experienced symptoms of GD as a teenager. However, when he lived at home with his parents, gaming was not a problem because his parents had some control over his gaming. The peak of the problem occurred at the beginning of his university years, after he had moved out of his parents’ home. During the first semester, he procrastinated a lot and experienced a relationship breakup. At that time, he was playing games for up to 16 hours a day. At the time of data collection, GD symptoms remained a challenge.
- P3 (male; in his 20s; university student) dropped out of university because of GD symptoms. He procrastinated a lot by playing games, which negatively affected his academic performance. This was the first time that he realized that he had no control over his gaming behavior. At the time of data collection, he was just beginning to change his gaming behavior.
- P4 (male; aged 12-16 years; high school student) played on PC and PlayStation mostly with friends he knew from offline life. In addition to gaming, he mentioned several analogue hobbies (eg, sports and music). He mentioned symptoms of GD when he was in elementary school. When his parents took away his cell phone, he played games on Nintendo Switch and hid it from his parents. At the time of data collection, he did not consider his gaming behavior problematic, although his mother did.
- P5 (male; aged 12-16 years; high school student) reported negative effects of gaming, such as not being on time for dinner or not studying for school. However, he himself did not seem to find his gaming problematic, seeing it primarily as an opportunity to have fun and be in contact with his friends. He would stop gaming if his friends asked him to play soccer with them and also mentioned various sports as analogue hobbies.
- P6 (male; aged 12-16 years; high school student) played games after stressful days because it relaxed him. He also played games when he was bored and when he needed to feel successful. At the time of data collection, he did not seem to consider his gaming as problematic, but there were symptoms of GD in the past. He had already reduced his gaming.

### Data Collection

Data collection was conducted between January 2023 and July 2023 by author SS, who was new to qualitative methods and IUD research at that time. Therefore, authors IB and BLG and other members of the project group, who have experience in qualitative research, IUD research, or IUD treatment, were involved in the design of the interview guides (Textbox 3). The project group consisted of male and female researchers between the ages of 20 and 60 years with backgrounds in psychology, medicine, health sciences, and communication sciences and with many years of experience in IUD research and therapy.

**Interview guide development.**

The interview guide was developed by translating the research interests into interview questions based on the “SPSS principle for creating interview guidelines” according to Helfferich [49]. In step 1, author SS collected as many questions as possible that could be of interest in relation to the research topic. In step 2, under the supervision of author BLG, SS gradually reduced the questions to those that were actually useful for answering the research questions. In a next round, and in exchange with BLG and the rest of the project group, SS performed steps 3 and 4 (sorting and subsuming). Finally, SS presented the interview guide to the Qualitative Methods Research Workshop at University Hospital Tübingen. After incorporating feedback from the workshop members and further consultation with the research team, SS finalized the interview guide.

The final interview guides, one for the focus group and one for the semistructured interviews, included the following topics: (1) participants’ self-introduction and their reasons for playing computer games, (2) experiences with (problematic) use of computer games and motivation to seek help, (3) experiences with strategies and offers of help that ameliorated problematic gaming behavior, (4) experience with online services in general, (5) user needs for a self-guided web-based intervention for gaming disorder, and (6) an open final question—“Is there anything else you would like to add at the end?”

The focus group lasted 98 minutes. It was moderated by author SS as a young male individual whom the participants might not associate with an authority figure, such as a parent or teacher. For this reason, author KUP, as the senior researcher who assisted and took field notes, deliberately remained in the background. Author SS introduced himself not only as a psychologist but also as a recreational gamer himself to establish a trusting relationship and invite participants to respond in detail. The semistructured interviews lasted 60 minutes each and were conducted by author SS alone. All interviews were audiotaped and transcribed. Participants in the semistructured interviews were familiar with author SS before the interviews. SS had no previous relationship with the focus group participants.

### Analysis

Data analysis followed the variant of qualitative content analysis by Kuckartz and Rädiker [47]. The pseudonymized transcripts were coded and analyzed by an experienced qualitative researcher (author BLG) combined with peer debriefings. The coding process was based on the principle of consensual coding [47] (ie, in this case, joint coding and joint discussions of codings and themes). It was decided against an approach that uses the principle of intercoder reliability as a validation strategy [50] because it is based on a quantitative paradigm [51] or postpositivist paradigm [52]. Rather, we followed a “Big Q” approach based on a qualitative paradigm [51] or interpretivist paradigm [52]. In this case, the validity of the analysis is not shown by the fact that different coders were able to agree on one “truth” but by allowing and discussing different perspectives on the data in exchange with other researchers. During the coding process, BLG repeatedly selected excerpts from all interview transcripts for joint coding. Data sessions took place with members of the research team (5 additional researchers participated: AS, KUP, IB, SH, and CL) and at the Qualitative Methods Research Workshop at University Hospital Tübingen, which is attended by an average of 5 to 8 researchers. In this way, a joint analysis took place to ensure the quality of the analysis process. This included the structuring and revision of the category system as well as the joint coding and discussing of rich and complex text passages. BLG also used the exchange to continually reflect and critically question her own perspective on the material. BLG wrote protocols for the analysis sessions and integrated the findings into the subsequent analysis process.

The coding process (see Textbox 4 [47] for details) was supported by the MAXQDA software (VERBI GmbH). Further in-depth and cross-case analyses were conducted using the coding tree (Multimedia Appendix 2) and theme matrices (see an example in Multimedia Appendix 3 [47]).

**Data analysis process. The data analysis in this study followed the qualitative content analysis by Kuckartz and Rädiker. It was slightly modified.**

- Familiarization with the material through repeated readings and initial comments
- Deductive development of major themes in advance based on research questions and interview guide
- Coding one transcript at a time guided by major themes and, during the coding process, deriving inductive codes (called subcategories in the coding tree) from the data while simultaneously coding the transcript
- Writing case summaries and memos
- Reorganizing the coding tree based on 3 levels of categories: superordinate categories, main categories, and subcategories; inductively generating the subcategories that contain detailed facets of the main category
- After coding all transcripts, creating theme matrices “cases times categories” for each superordinate category
- Coding the material again
- Comparisons within and across cases and further in-depth analysis using the theme matrices
- Identifying patterns and ambivalences in the data using the coding tree and theme matrices with repeated returns to the original data

## Results

### Overview

**Figure 1:**
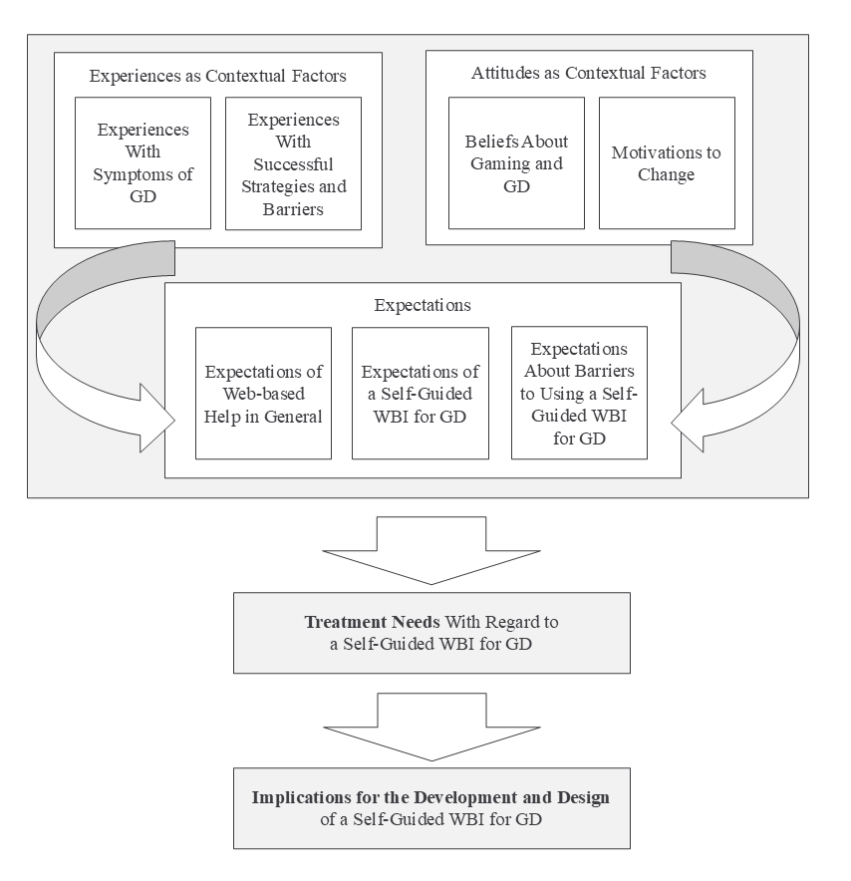
Operationalization of treatment needs in this study as major themes. GD: gaming disorder; WBI: web-based intervention.

This study aimed to understand the treatment needs of young people with GD in relation to the development of a self-guided WBI for GD. However, during the analysis, it became clear that focusing solely on explicitly stated user needs would not be sufficient. In analyzing the data, it became necessary to differentiate the treatment needs, which resulted in the following overarching themes: experiences and attitudes as contextual factors, as well as specific expectations of a WBI, assuming that contextual factors, in turn, influence expectations of a GD intervention (Figure 1). This is in line with the International Organization for Standardization 9241-210:2010 standard, which states that human-centered design activities should also include understanding and specifying the context of use [53]. In the following sections, we first present major themes that involve contextual factors: (1) experience with GD symptoms, (2) experience with successful strategies and barriers, (3) experience with web-based support in general, (4) beliefs about gaming and GD, and (5) motivations for change. We then present the major themes: (6) expectations of a self-guided WBI in general, (7) expectations of a self-guided WBI for GD, and (8) expected barriers to its use. Direct quotes were translated from German and edited for clarity (ie, condensed to the most relevant parts or edited if jargon was used or if the original sentence was not grammatically correct).

### Experiences as Contextual Factors

#### Experiences With Symptoms of GD

All participants had been gaming for many years, had experienced (temporary) symptoms of GD, and had experienced negative consequences of gaming. The participants identified needs that are met through gaming, as well as risk situations.

Several participants reported negative social consequences, such as a negative impact on school, studies, and family life (P1, P3, P5, and P6). Some participants reported unpleasant emotional consequences of gaming (P1, P3, P5, and P6), such as loss of control, anger after failing in the game, and feeling empty after finishing the game. Physical consequences were also reported, such as experiencing a “hot head” from playing for too long:

But if you play too much, you can get stupid...Because then I’ve noticed that my head gets really hot.

P6; aged 12-16 years

Nevertheless, regardless of the negative effects, gaming and even symptoms of GD seemed to meet certain needs. All participants used gaming to regulate unpleasant emotional states, such as dissatisfaction, boredom, or stress. In addition, several participants used gaming to create positive emotional states, such as feeling successful, feeling stimulated, having fun, experiencing adventure, and as a balance after a day full of obligations (P3, P4, P5, and P6). Another important aspect was the regulation of social needs through gaming—for P4 and P5, gaming provided an opportunity to meet friends from school. On the other hand, for P3, one of the older participants, gaming was about “finally” having time for himself (ie, distancing oneself from social contacts).

In addition, the data indicated various risk situations in which participants showed symptoms of GD. For older participants, the importance of transitional periods as risk situations became clear. For P1, P2, and P3, GD symptoms appeared at the beginning of university combined with moving out of the parental home (which led to the loss of parental control). Other factors contributing to the development of GD symptoms included study-related stress situations, such as exams, a general lack of structure in daily life, and personal crises, such as separation from a romantic partner. In addition, P1, P2, and P3 reflected that their gaming had long been an invisible problem that developed unnoticed until they moved out:

P1: I think it was in 9th or 10th grade when I first started thinking about it. StarCraft II was a problem at that time. But it never had any direct consequences because I was doing well in school and getting good grades. So my parents were not worried...And up until then, I didn’t really have much of a social environment that I would have lost touch with. So, it...lay dormant for quite a while, I’d say...without it hurting.

P3: Yes...It lies dormant for a couple of years. Until you get out of school. And then it really hits you...during your studies. I also feel like it starts earlier and then it boils over.

Focus group; participants in their 20s

This is consistent with the experiences of the younger participants (P4, P5, and P6), who were still living with their parents at the time of the study. They also showed uncontrolled gaming, especially when parental control was lacking, such as when parents were absent. Other risk factors affecting the adolescents were sleep problems (ie, playing because of sleeplessness) and peer groups that also played a lot.

#### Experiences With Successful Strategies and Barriers

All participants reported successful use of strategies to cope with GD symptoms outside of the internet, as well as barriers to using these strategies.

 All participants seemed to benefit from external stabilizers, such as parental control, daily routines, and support group meetings. Strategies also included reducing gaming or taking a break from gaming (P1, P2, P3, and P6), such as uninstalling the game, deleting the account, or using an app blocker. The older participants (P1, P2, and P3) mentioned different variations of self-reflection as a strategy, such as reflecting on general life satisfaction. The strategy of taking personal responsibility was also important (P2, P4, P5, and P6; eg, making rules for oneself). On the other hand, not expecting too much of oneself and avoiding extremes (P4, P5, and P6) was also mentioned as a strategy, for example, not imposing excessive restrictions on oneself and allowing oneself a defined daily dose of gaming. For the older participants and P5, alternative activities were important, especially sports and activities that were fun or provided a “high” (see the Beliefs About Gaming and GD section).

However, participants also experienced barriers when using strategies. In particular, the barrier of too much pressure should be noted (P1, P2, P3, and P6) as opposed to the barrier of not enough pressure (P1, P2, P3, P4, and P5). On the one hand, the barrier of too much pressure (see the following quotation) referred to strategies that were too strict or overwhelming and, therefore, actually prevented addressing GD symptoms, such as stopping gaming overnight without building alternatives in parallel, trying to cope with GD alone, using treatment tools that were not applicable, and making agreements with others to reduce or stop gaming (P1, P2, P3, and P6). On the other hand, the barrier of not enough pressure also prevented them from addressing GD symptoms, including not being sufficiently aware of the GD symptoms and, therefore, not feeling the need for help or negative consequences not being “painful” enough:

P2: If you promise your parents that you won’t play this week and then you don’t keep the promise because the pressure of addiction is too great, then it’s rather counterproductive [to make agreements at all]. Then I feel like I’ve disappointed my parents...I’m so ashamed that I feel...even worse...And then I actually play even more.

P1: Right, then you might want to drown this feeling of shame, and then it gets worse.

P2: Yes, yes, exactly.

P3: Agreements in general never helped me either...My mother once wanted to make a plan with me. I stuck to the plan for one day at most, and then I threw it out again.

P2: It’s the same with making plans with oneself.

P3: Making plans with oneself is also terrible. It doesn’t work either.

Focus group; participants in their 20s

### Attitudes as Contextual Factors

#### Beliefs About Gaming and GD

Participants held certain beliefs about gaming and GD. Of particular relevance were the beliefs of lack of agency versus agency and the dopamine hypothesis.

Regarding the lack of agency belief, the older participants seemed to be partially convinced that they had no agency in their GD (P1, P2, P3, and P6). This included the belief that there was a “gamer gene” that could be activated repeatedly. In this case, the participants seemed to experience their game as an overwhelming force rather than as a behavior of their own. The game had agency, not the player. Therefore, when using a self-guided WBI for GD, technological barriers to the game would be necessary:

I need some kind of [separate] electronic device on which I can do this online training...A blocker while I’m using this training, so that I don’t just drop out and end up in a game.

P1; focus group; participants in their 20s

In addition, P2 and P3 believed that proper parental behavior regarding their children’s media use was crucial in preventing the child from later developing GD. This can be interpreted as parents being responsible for later GD, not the gamers themselves. P6 believed that too much parental control could lead to the development of addiction:

Parents sometimes create the addiction themselves by letting you play so little.

P6; aged 12-16 years

In this case, the agency lay with the parents and not with the gamers. However, at the same time, many participants emphasized the importance of personal responsibility, which in turn implies having agency (P2, P3, P4, and P6).

This belief was evident in both older and younger participants, for example, the belief that a person has to really want something; otherwise, nothing will change. P2 said that it was not the games themselves that caused the problem but people’s thoughtless use of them. These beliefs implied that it was not the game that had the power but the gamer. It is important to note that this is not an “either/or” situation; rather, participants seemed to feel both a lack of agency on the one hand and full responsibility for their behavior on the other.

Another relevant belief was the so-called dopamine hypothesis (P1, P2, P3, and P6). This belief implied that gaming was associated with rewarding effects and that the “highs” experienced in gaming were difficult to find elsewhere. Therefore, if the alternative activity to gaming was not sufficiently attractive, one would inevitably play again. Only those activities that provided a “kick” were worthwhile:

I realized that doing sports helps me very little. Especially weight training doesn’t help me because I don’t get anything out of it. It’s all about dopamine, and I just don’t get any dopamine from it.

P3; participant in his 20s

Belief in the dopamine hypothesis seemed to influence expectations regarding the design of a WBI for GD—if the WBI did not provide the necessary rewarding experience, it might even be a risk factor as switching to the game was only a click away:

And when the game is only 2 clicks away, and your dopamine is only 2 clicks away, you end up in the game very quickly.

P3; participant in his 20s

In this case, the dopamine hypothesis theme can be seen in connection with the lack of agency theme—the rewarding effect had ultimate power over the player.

#### Motivations to Change

Participants mentioned several motivational factors as to why they had accepted help regarding their GD symptoms in the past: the opportunity to connect with others; autonomy and making one’s own decisions; positive, solution-oriented attitudes; and emotional distress.

The ability to connect with others was an important motivational factor for most participants (P1, P2, P3, P5, and P6). For example, they felt motivated when another person gently approached them about their GD, when they could share general life experiences, and when they received appreciative feedback from others about their progress. Connecting with others seemed to be particularly motivating when a participant was overly self-critical:

If you are very, very negative in your self-evaluation, sometimes an outside influence is very good...You think: I’ve just been gaming here all week. And then somebody else tells you: Yeah, but...first of all, you came back [to the support group]. And secondly, you did this and that differently that day.

P1; participant in his 20s

Another motivating factor was autonomy and making one’s own decisions (P2, P5, and P6). This included, for example, being able to decide whether an offer of help was right or develop one’s own solutions. P6 (aged 12-16 years) in particular seemed to be someone who was motivated by a sense of autonomy. Gaining autonomy and developing one’s own identity is important during adolescence. Therefore, it seemed to be very important for P6 to be actively involved in decisions—arrangements about gaming between him and his parents had to be workable and understandable, and he wanted to be in agreement with them. In addition, he did not like to be controlled when following the rules; instead, agreements should be based on trust.

This was consistent with another motivating factor: offering help in a positive, solution-oriented attitude rather than a narrow focus on the problem (P1, P2, P3, P5, and P6). Almost all participants felt motivated when an offer of help included the development of attractive alternatives and goals and encouragement to change by focusing on positive prospects and promises of success. Interestingly, P6 needed to be first shaken and startled and then presented with a solution:

In my case, it...should scare me a little bit. That is how advertising works: If you want to get money from customers, you have to scare them a little [laughs]. Then you have to offer a solution, and then [the customer] clicks on it.

P6; aged 12-16 years

In this case, P6 combined the solution-oriented motivating factor with emotional distress as a motivating factor. Emotional distress was especially reported by older participants as the initial motivation for seeking help (P1, P2, and P3), for example, caused by the negative impact of gaming on academic performance. P1 reported that the experience of losing control over gaming led to feelings of shame, which motivated him to seek help. However, at another point in the focus group, shame was mentioned as a barrier. Emotional distress seemed to work in both ways: it could motivate change, but it could also lead to the avoidance of change.

### Expectations

#### Expectations of Web-Based Help in General

Interestingly, only 1 participant, P5, had experience with web-based help in the form of a self-guided online media literacy training. The following characteristics of a self-guided WBI were derived from P5’s positive and negative experiences with this type of web-based help:

- Quality design—the WBI should be attractively designed. Content should be presented in a variety of ways, for example, not only textual content but also playful elements.
- Comprehensibility—the WBI should be easy to understand, for example, by providing a guide with explanatory elements on how to use the WBI and providing age-appropriate content while avoiding unfamiliar terms.
- Challenging—the WBI should encourage self-reflection, for example, by offering relevant new information or providing a final quiz at the end of a unit to check the user’s learning status.
- Autonomy enablement—the user should be able to actively search for relevant content. The WBI should be accessible through a variety of devices.

These ideal characteristics of a self-guided WBI in general came from only 1 participant. However, they were formulated in such a nuanced way and related so well to the experiences described in the following section that P5’s evaluations are presented here anyway.

#### Expectations of a Self-Guided WBI for GD

The participants expressed a variety of expectations for a potential self-guided WBI for GD.

In terms of content, all participants expected the self-guided WBI to connect with their everyday life, for example, by referring to the lived experiences of real people rather than to scientific theory:

Interviewer: For example, what would an online training absolutely have to do or include for you to say I’m going to take a look at it?

P6: It would have to appeal to the person; it would have to come from experience; and it would definitely have to be logical. And it would have to be genuine...You know, a study can mean a lot of things, but...what worked in the end is what worked and not what the study says.

Aged 12-16 years

In addition, a self-guided WBI should provide content that invites self-reflection (P1, P2, P3, P4, and P5), for example, by asking questions about gaming behavior and tracking it over time or by inviting nonjudgmental reflection on negative consequences. All adolescent participants wanted the WBI to offer personal counseling (P4, P5, and P6), whereas 2 of the older participants (P1 and P2) saw the self-guided WBI as a bridge to local counseling. Again, it was P5 who had detailed ideas when asked about the content design of a self-guided WBI for GD:

I can imagine that you can...choose how old you are...You can put in different hobbies or what you do. Then you can put in what your schedule or a typical week looks like, when you play, and how much you play. Every 3 or 4 days you can go back to the website and put in how long you’ve been playing, what you’ve been playing, and what you’ve been doing. At the end of the week, you can discuss with someone how long you’ve been playing. And then they can have a look at it, and then you can make a plan for what you want to do.

P5; aged 12-16 years

P4 also provided detailed information. The WBI should (1) discuss the initial gaming behavior and develop a plan for change, (2) facilitate the solution-oriented development of a vision or goal, (3) keep in touch with the user and ask about the status of the change, (4) allow for tracking of change implementation, (5) provide a reminder function to limit play time, and (6) suggest motivating and rewarding alternatives to make it easier to stop playing.

What can be seen here is the users’ need for a self-guided WBI that repeatedly communicates with them, similar to a human counselor or therapist.

In terms of structure, participants wanted a self-guided WBI to be flexibly accessible at a low threshold (P2, P4, P5, and P6). For example, it should be usable on all devices, browser based, and spontaneously accessible in critical situations without a time-consuming registration process. Participants’ statements about the desired duration and frequency of use varied—P1, P2, P3, and P5 expected to use a WBI for 15 to 30 minutes per day, whereas P6 expected to use a WBI only once for 30 minutes and the other participants would use it several times. P4, on the other hand, only wanted short videos of 3 to 5 minutes:

Interviewer: What do you think? Fifteen minutes, half an hour, an hour? How long would it take to watch videos to get back on track?

P4: I think the TikTok video I watched was 2 or 3 minutes long. But it was not unnecessary talk; it was real words. Nice and clear, all well-spoken. Right. And I really got it. It’s true. And it doesn’t take a long time. But the longer you think about it, the better it is. If you get it right, then you need...5 minutes to look at it. And then you sit down somewhere and...think about it a little bit. And in this case, I realized: Yeah, that’s right. And then I got a better handle on this and that.

Aged 12-16 years

Regarding optimal promotion, almost all participants (P2, P3, P4, P5, and P6) expected video-based advertising to be easy to find on social networks, especially YouTube and TikTok. One expectation was that the WBI would be promoted by social media influencers (P1, P2, P3, and P6) or by a therapist (P6).

#### Expectations About Barriers to Using a Self-Guided WBI for GD

Participants mentioned several possible barriers if they were to actually use a self-guided WBI for GD: negative framing, lack of differentiation, no reflection of the reality of the user’s life, use of gamification elements, and focus only on digital help.

Negative framing was seen as a barrier by all participants. For example, some participants found it critical to confront users with a negative assessment of their gaming behavior. The use of the term *addiction* was also viewed critically by all participants. Some felt that being labeled as addicts could be perceived as an attack or devaluation. P1 found it problematic to title the WBI using the term *addiction* as some of those affected were not yet aware that they had a problem and, therefore, would not feel addressed by it. Therefore, a neutral or solution-oriented title such as “media training” or “screen time reduction training” was suggested. The negative framing barrier shows that, from the perspective of potential users, a self-guided WBI should avoid echoing the familiar restrictions and prohibitions that young users already hear all the time, for example, from their parents:

When you google something like that, the first thing you find is always the parents, who...say: listen to your father, and this and that...It’s the same thing you hear from your parents. It’s just annoying...Let people talk who have already been through it, let them talk, that is much more pleasant and refreshing.

P6; aged 12-16 years

A lack of differentiation was also seen as a barrier (P2, P3, P4, P5, and P6). This included, for example, too much standardization of the WBI content, such as presenting a one-size-fits-all solution plan instead of responding individually to users, or focusing solely on the goal of stopping gaming and demonizing gaming across the board. A boring design that repeatedly presented content in the same manner was also considered a barrier.

Another barrier mentioned was the WBI not reflecting the reality of the user’s life (P1, P2, P3, P5, and P6), for example, by mainly reflecting the perspective of nongamers, parents, or scientists. It would also be considered problematic if the intervention was mainly based on textual content and if it could only be used on nonmobile devices.

In contrast to the barriers described previously, the barrier of using gamification elements stood out. One might think that gamification elements would be seen as helpful to connect with the users’ world. However, some participants found them problematic (P1, P3, and P6). At the same time (eg, regarding the dopamine hypothesis), gamification elements are a good way to make a WBI attractive. This shows that gamification elements were seen as both beneficial and risky. P6 felt that the use of educational games in a WBI could again lead to GD:

That sounds stupid. I don’t know, I can’t really imagine a game like that...So I play this [educational] game. But I would end up playing again. I would tell myself that I’m playing less, but in reality, I’m playing again. So I’d have invested 2 hours in this [educational] game, whereas I used to invest 6 hours in the real game. So, 4 hours in this game [the real game] and 2 hours in that game [the educational game]. And then what? Well, it’s not much use.

P6; aged 12-16 years

Finally, focusing only on a self-guided WBI was also seen as a barrier to addressing GD symptoms (P2 and P4). For example, instead of offering only an online chat with other users affected by GD, the user should be guided into conversations with people outside the internet.

## Discussion

### Principal Findings

#### Overview

This qualitative study took initial exploratory steps to identify user needs regarding the characteristics of a self-guided WBI for German adolescents and young adults with GD. Changing a GD or any other harmful behavior is a major challenge. Therefore, ambivalence toward change is a key issue that any intervention should address, especially a self-guided WBI that can only provide standardized, “fixed” content. The following ambivalent treatment needs represent, at a further level of abstraction, a condensed version of the ambivalences that were presented in the Results section. We then discuss the implications of ambivalence for the design of a WBI for GD. The results indicate that, although a self-guided WBI for GD can be considered a low-threshold treatment option, its development is challenging. A standardized, self-guided WBI must find ways to address the complexity of young people’s experiences of GD and their internal ambivalences during a change process. The content of the WBI, as well as its structure, storyline, protagonists, tasks, and language, must be adapted to the fact that facing and changing a possible GD can be associated with internal struggles for the user.

#### Ambivalent Treatment Needs

##### “Be Entertaining but Not Triggering”

All participants expressed the need for a WBI to be attractively designed. This finding is consistent with the observations by Bosworth et al [54], who used the System Usability Scale [55] and qualitative interviews to explore user requirements and design preferences during the development of a mobile health app for adolescents. For example, they found that “adolescents prefer vibrant colors, modern, easy-to-use interface, gamification and rewards, customizable and personalized, simple and mature graphics. Adolescents were especially motivated by gamification techniques in maintaining their interest in the application and their health behavior goals” [54]. Findings such as these are the reason gamification elements were integrated into the design of the Breaking the Game WBI. However, the results of our study showed that some participants were concerned that gamification elements in the WBI would trigger GD symptoms. In this respect, the results of our study contrast with those of the study by Bosworth et al [54], who generalize their findings in a way that does not reflect adolescents with GD. The results of the study presented in this paper draw attention to the risk of a “too attractive” design in the development of self-guided WBIs for GD. This has not been thoroughly reflected in previous research on WBI development as the WBI being too attractive is a specific problem for people with GD or other IUDs, and there is still a lack of research literature reflecting on the development of a self-guided WBI for GD, especially with regard to design. Studies of web-based treatment options for people with GD or other IUDs still focus too much on the therapeutic concept and technological advantages (eg, accessibility and anonymity) rather than on the way in which the treatment option is presented [26].

In this context, it is also necessary to consider users’ beliefs about their problem and possible solutions that might influence the use of a WBI. For example, the idea that alternatives to a game should be as rewarding as the game itself was mentioned by several participants in this study. From the research team’s perspective, a self-guided WBI should, while nicely designed as a virtual place to return to, also address the question of whether it is helpful to constantly seek reward and comfort. The dopamine hypothesis should be addressed in the WBI, for example, by informing users that the brain needs time to adjust to lower levels of dopamine and that it may be necessary to initially forgo the “high” in alternative activities.

##### “I Want to Change, but Change Means Stress, and I Cannot Handle Stress”

The data from this study showed that gaming, even symptoms of a GD, could meet important emotional self-regulation needs, such as stress management or generating positive emotional states. This is consistent with the findings of Rollo et al [56] that internet use can meet important needs of young people that are not met in the offline world. It is also consistent with the findings of other studies linking the occurrence and persistence of IUD or GD symptoms to deficits in emotion regulation [57,58]. This means that using a WBI for GD may feel risky. Young people with GD may be used to suppressing feelings, avoiding direct confrontation with problems [59], and regulating unpleasant feelings through gaming [60]. Therefore, a self-guided WBI for GD must be designed to encourage users to reflect on the needs they are satisfying through gaming. In addition, users should be provided with alternative emotion regulation strategies and shown new ways of satisfying the needs that are currently mainly met through computer games. This is in line with the work by Gurdal et al [61], who concluded that adolescents with GD find it helpful to understand the reasons for their problematic gaming. This also means that IUD or GD interventions should not focus solely on controlling or reducing internet use [56].

However, there is a gap in knowledge on how to design a WBI for GD to help users who are stressed with their change process. Guided WBIs for GD and IUD already include change-sensitive techniques, such as motivational interviewing in their concept [11], but there is still a lot to learn regarding self-guided WBIs for GD because there is no counselor or therapist to apply motivational interviewing techniques.

##### “Help Me With My Gaming Problem but Don’t Call Me an Addict”

The comments of some study participants showed that too much pressure was a significant barrier to changing problematic gaming behavior. From their perspective, a self-guided WBI should not negatively frame the user’s gaming behavior, for example, by calling it an “addiction.” It should also avoid creating too much pressure by repeating parental restrictions. At the same time, the lack of pressure seemed to be an equally important barrier in addressing GD symptoms.

For the development of a self-guided WBI, this means finding a good balance between demonstrating the negative consequences of GD symptoms and simultaneously avoiding high demands and rigidly formulated strategies. Instead, the WBI could make it transparent to the user that a change in gaming behavior may cause temporary stress and provide coping strategies. The WBI should not say that people *are* addicts but that they *have* an addiction—and they can cope with it, even if it is hard to imagine at first. Stigmatization or self-stigmatization can have a negative impact on mental health patients’ self-efficacy and adherence to treatment [62,63]. Therefore, the importance of wording is significant in the development of a self-guided WBI. As logging out is only a click away, balanced language is critical.

##### “The Game Is Not the Real Problem, My Life Is”

The data from this study showed that life transitions, such as leaving home, were experienced as challenging, which, in turn, increased the risk of developing a GD. A self-guided WBI for GD should take this into account—in addition to their GD, users may be experiencing a general life crisis. Therefore, it is not enough to focus on GD alone. The WBI should also address other problems in the user’s life, such as stress at school, university, and work, as well as social problems. This is in line with the study by Rollo et al [56], which explored the needs of adolescents in relation to the prevention of problematic internet use. From their perspective, interventions should not be limited to the level of the individual but should take a broader perspective by reflecting on the social environment [56].

##### “I Resent External Control, but I Also Need It”

The results of this study suggest that an intervention that lacks freedom of choice and autonomy may discourage coping with GD symptoms. However, at the same time, external control, for example, from parents, worked as a helpful strategy for participants, preventing escalation of gaming behavior for many years. Technology also seemed to provide external control. For example, app blockers were mentioned as helpful for several participants. Does this mean that technology is allowed to control gaming behavior, whereas people are not? The key point seems to be that it was the participants’ choice to allow external control in the form of technology into their lives, whereas parental control was not a choice but was imposed on them. This suggests that the need for external control does not necessarily contradict the need for autonomy as long as it is based on free choice. However, age-related differences should be taken into account. From a developmental perspective, it can be assumed that adolescents are less able to control their gaming than young adults [64]. At the same time, adolescents may be driven by a strong need for autonomy.

For the development of a self-guided WBI, this means finding the right amount of guidance while considering different age-related autonomy needs. The WBI should allow for autonomy in its use while providing a clear structure and social support, such as a forum. Language and wording should address the target group in a way that does not patronize them but invites them to participate. Control tools, such as tracking gaming behavior, should be offered in a positive and inviting way.

#### Integrating Ambivalence Into WBI Design

Ambivalence about changing a GD (or any other problem behavior) is very normal. It is also a significant challenge in offline face-to-face therapy. However, as a self-guided WBI cannot respond to the user’s ambivalence as flexibly as a human therapist can, it must find other ways to accommodate the user’s ambivalence.

One basic attitude that a self-guided WBI can adopt is to communicate it openly to the user, such as in the following way: “We recognize that certain aspects may make it difficult for you to change. You may sometimes feel ambivalent and ask yourself: Do I really want to change my gaming? While another part of you may actually want to change. We believe that change is possible, and we want to help you get there.” To be successful, a self-guided WBI, just like a face-to-face therapy, must be able to oscillate between the ambivalent poles of the target group.

The technique of motivational interviewing can be considered the gold standard in addiction treatment and has been used in IUD WBIs with human therapists with the specific goal of resolving ambivalence and building motivation to change [11,12]. A self-guided WBI may not be able to replace face-to-face motivational interviewing. However, it can incorporate design elements based on motivational interviewing principles to promote user compliance.

#### Accepting and Expanding the Boundaries of Self-Guided WBIs

As mentioned previously, for some people with GD symptoms, a self-guided WBI may not be sufficient. In some cases, it may be better to seek more individualized help to address the underlying problem for which gaming may be a coping strategy. Some of the study participants expressed a clear need for a more personalized approach, suggesting that the WBI should include counseling by a human therapist.

However, studies show that a self-guided WBI can be designed to strengthen the bond between the user and the treatment program. In a scoping review, Scholten et al [33] investigated how embodied conversational agents can support users of self-guided WBIs and contribute to adherence. Therefore, the effectiveness of artificial intelligence, such as conversational chatbots and dialogue systems, is currently the subject of intense research [65]. Holter et al [66] investigated the person-to-program alliance between users and self-guided WBIs. The authors found 3 typical relational patterns. They showed that some of the interviewed participants developed semisocial interactions and even semisocial relationships with the digital program [66].

### Limitations and Perspectives

This study has limitations that must be noted.

The results are based on a small sample of 6 young gamers. As this study revealed heterogeneous and sometimes contradictory user needs, it was sufficient to provide guidance for the development of the WBI Breaking the Game for a German target population. It revealed a variety of experiences and perspectives of young people. The research group was successful in integrating different age groups and educational backgrounds. The triangulation of different data collection methods depending on the age group (a focus group with the older participants and individual interviews with the younger participants) contributed to the quality of the data. However, the sample of this study could only provide an initial basis for exploring and identifying relevant aspects that could be built upon in a larger study (eg, to derive quantitative items). Follow-up studies are needed to deepen and generalize the initial findings of our study.

In addition to the limited sample size, it is critical to note that the sample consisted entirely of male participants, although studies have shown that young women can also be affected by GD [67,68]. A German prevalence study showed that boys were twice as likely as girls to be affected by GD in 2023 [7]. In our view, this reflects the limited perspective of the research field and the continuing gap in care in Germany, which is reflected in the fact that women with GD continue to be significantly less represented in the health care system than men. It is possible that only male individuals were recruited because of the focus on the health care system. The support group consisted of male participants only. Therefore, the sample of this study should be viewed critically as it reproduces the research gap on GD in women. It may also have been an obstacle that SS, as a male researcher, recruited participants in the outpatient clinic. This may have made it less likely for female participants to take part. This limitation of our study demonstrates the importance of sensitivity to diversity but also diversity among researchers themselves to generate findings that reflect the diversity of reality. It would be important for researchers to be more aware of their field affiliation and actively seek participants outside their field.

Another limitation relates to the research design and the level of participation. During the analysis, it was interesting to observe that some of the participants’ statements were particularly irritating to the members of the research team responsible for developing the WBI. “What did he mean by that?” was a common question. This suggests that additional participatory elements would have been useful. For example, an additional group discussion or member-checking workshop could have been conducted with the participants after the analysis, with questions played back to the participants to validate or clarify their meaning. We believe that a strength of this study is that, by including the end-user perspective in the development of the Breaking the Game WBI, the project was in line with the World Health Organization’s advocacy for collaborative and responsible development of youth-centered digital health interventions. The World Health Organization considers it as a no-go to involve young people “only at the end of the process or only at one stage of the process” [69]. However, one might ask what other collaborative formats might be possible besides qualitative focus groups and interviews. This is, of course, also a question of resources, which would need to be planned for at the outset. The World Health Organization framework states that a part of the process should involve young people as cocreators. “They are the experts on what health information young people need and what technology young people are using” [69]. Their involvement “can range from having several young people engaged in every aspect of planning, development and implementation to having a youth advisory board to consult throughout the process” [69].

The financial and time constraints of research projects may make this difficult to achieve. Nevertheless, a more intensive involvement of end users in the development of the self-guided WBI would have been instructive and should be considered in future research projects.

### Conclusions

Given the diversity of expectations expressed by a group of 6 young people, it can be assumed that it is impossible to design a self-guided WBI that meets all user needs. However, it must not be the goal to map user needs 1:1 in a WBI. On the contrary, ambivalence of needs in the face of a possible GD should be seen as a normal part of a change process and, therefore, actively integrated into the WBI concept and storyline. A self-guided WBI for GD should make the consideration and management of conflicting user needs its main concept—this is what the reality of people with GD is all about. In the development of the Breaking the Game WBI, for example, this was taken into account by having the protagonist himself oscillate between the motivation to change and the urge to return to the game in stressful situations. This is not presented as a problem but as his personal way of getting to know himself better, which turns out to be the basis for a sustainable change toward healthier internet use.

## Appendix

Multimedia Appendix 1 COREQ checklist.

Multimedia Appendix 2 Coding tree.

Multimedia Appendix 3 Theme matrix example.

## Abbreviations

COREQ: Consolidated Criteria for Reporting Qualitative Research
GD: gaming disorder
IUD: internet use disorder
WBI: web-based intervention

## Data Availability

The datasets generated or analyzed during this study are available from the corresponding author on reasonable request.

## References

[1] (2024). 6C51 Gaming disorder: ICD-11 for mortality and morbidity statistics. World Health Organization.

[2] Dreier M, Wölfling K, Beiglböck W, Gottwald-Nathaniel G, Preinsperger W, Scheibenbogen O (2023). The treatment of computer gaming and internet use disorder in times of digitalized addiction treatment. Addiction Treatment and Digitalization: Addiction Prevention and Addiction Therapy Between Human Encounters and Virtual Reality.

[3] Han TS, Cho H, Sung D, Park M (2022). A systematic review of the impact of COVID-19 on the game addiction of children and adolescents. Front Psychiatry.

[4] Montag C, Pontes HM, Kannen C, Rozgonjuk D, Brandt D, Bischof A, Salbach H, Mößle T, Wölfling K, Rumpf H (2024). Examining the interplay between internet use disorder tendencies and well-being in relation to sofalizing during the COVID-19 pandemic. Compr Psychiatry.

[5] Pape M, Geisler BL, Cornelsen L, Bottel L, Te Wildt BT, Dreier M, Herpertz S, Dieris-Hirche J (2023). A short-term manual for webcam-based telemedicine treatment of internet use disorders. Front Psychiatry.

[6] (2020). Mediensucht 2020 - Gaming und Social Media in Zeiten von Corona. DAK-Längsschnittstudie: Befragung von Kindern, Jugendlichen (12-17 Jahre) und deren Eltern. DAK-Gesundheit.

[7] (2024). Problematische Mediennutzung im Kindes- und Jugendalter in der post-pandemischen phase: Ergebnisbericht 2023. DAK-Gesundheit.

[8] Horvath KJ, Ecklund AM, Hunt SL, Nelson TF, Toomey TL (2015). Developing Internet-based health interventions: a guide for public health researchers and practitioners. J Med Internet Res.

[9] Bücker L, Gehlenborg J, Moritz S, Westermann S (2021). A randomized controlled trial on a self-guided internet-based intervention for gambling problems. Sci Rep.

[10] Baumeister H, Reichler L, Munzinger M, Lin J (2014). The impact of guidance on Internet-based mental health interventions — a systematic review. Internet Interv.

[11] Dieris-Hirche J, Bottel L, Basten J, Pape M, Timmesfeld N, Te Wildt BT, Geisler BL, Wölfling K, Henningsen P, Beutel M, Neumann A, Niemann A, Beckers R, Herpertz S, OMPRIS Study Group (2023). Efficacy of a short-term webcam-based telemedicine treatment of internet use disorders (OMPRIS): a multicentre, prospective, single-blind, randomised, clinical trial. EClinicalMedicine.

[12] Bottel L, Brand M, Dieris-Hirche J, Herpertz S, Timmesfeld N, Te Wildt BT (2021). Efficacy of short-term telemedicine motivation-based intervention for individuals with internet use disorder - a pilot-study. J Behav Addict.

[13] Grüning DJ, Riedel F, Lorenz-Spreen P (2023). Directing smartphone use through the self-nudge app one sec. Proc Natl Acad Sci U S A.

[14] Bischof A, Brandt D, Schlossarek S, Vens M, Rozgonjuk D, Wernicke J, Kannen C, Wölfling K, Dreier M, Salbach H, Basenach L, Mößle T, Olbrich D, König I, Borgwardt S, Montag C, Rumpf H (2022). Study protocol for a randomised controlled trial of an e-health stepped care approach for the treatment of internet use disorders versus a placebo condition: the SCAPIT study. BMJ Open.

[15] Jeon M, Lee MS, Yoon JY, Bhang SY (2022). Mental health literacy of internet gaming disorder and problematic smartphone use among Korean teenagers. PLoS One.

[16] Granero R, Fernández-Aranda F, Castro-Calvo J, Billieux J, Valero-Solís S, Mora-Maltas B, Rivas-Pérez S, Valenciano-Mendoza E, Del Pino-Gutiérrez A, Gómez-Peña M, Moragas L, Baenas I, Mena-Moreno T, Casalé-Salayet G, Codina E, González-Bueso V, Santamaría JJ, Baño M, Menchón JM, Jiménez-Murcia S (2021). Subtyping treatment-seeking gaming disorder patients. Addict Behav.

[17] Torres-Rodríguez A, Griffiths MD, Carbonell X, Oberst U (2018). Internet gaming disorder in adolescence: psychological characteristics of a clinical sample. J Behav Addict.

[18] Gao YX, Wang JY, Dong GH (2022). The prevalence and possible risk factors of internet gaming disorder among adolescents and young adults: systematic reviews and meta-analyses. J Psychiatr Res.

[19] Schneider LA, King DL, Delfabbro PH (2017). Family factors in adolescent problematic internet gaming: a systematic review. J Behav Addict.

[20] Brandhorst I, Renner T, Barth GM (2021). Parental factors in internet and computer game addiction in adolescence: an overview. Z Kinder Jugendpsychiatr Psychother.

[21] Dong GH, Potenza MN (2022). Considering gender differences in the study and treatment of internet gaming disorder. J Psychiatr Res.

[22] André F, Broman N, Håkansson A, Claesdotter-Knutsson E (2020). Gaming addiction, problematic gaming and engaged gaming - prevalence and associated characteristics. Addict Behav Rep.

[23] Sweeney GM, Donovan CL, March S, Forbes Y (2019). Logging into therapy: adolescent perceptions of online therapies for mental health problems. Internet Interv.

[24] Howes ST, Gorey KM, Charron CM (2021). Relative effectiveness of online cognitive behavioural therapy with anxious or depressed young people: rapid review and meta-analysis. Aust Soc Work.

[25] Domhardt M, Ebert D, Baumeister H, Fegert J, Eggers C, Resch F (2021). Internet and mobile-based interventions in children and adolescents. Psychiatry and Psychotherapy of Children and Adolescents.

[26] Gorowska M, Tokarska K, Zhou X, Gola MK, Li Y (2022). Novel approaches for treating internet gaming disorder: a review of technology-based interventions. Compr Psychiatry.

[27] Balhara YP, Sarkar S, Laspal N, Bhargava R, Yadav Z (2023). A randomized controlled trial to assess effectiveness of GamE- an e-Health intervention to self-manage gaming with an aim to prevent gaming disorder. Asian J Psychiatr.

[28] Sioni SR, Burleson MH, Bekerian DA (2017). Internet gaming disorder: social phobia and identifying with your virtual self. Comput Human Behav.

[29] Gulliver A, Calear AL, Sunderland M, Kay-Lambkin F, Farrer LM, Banfield M, Batterham PJ (2020). Consumer-guided development of an engagement-facilitation intervention for increasing uptake and adherence for self-guided web-based mental health programs: focus groups and online evaluation survey. JMIR Form Res.

[30] Karyotaki E, Riper H, Twisk J, Hoogendoorn A, Kleiboer A, Mira A, Mackinnon A, Meyer B, Botella C, Littlewood E, Andersson G, Christensen H, Klein JP, Schröder J, Bretón-López J, Scheider J, Griffiths K, Farrer L, Huibers MJ, Phillips R, Gilbody S, Moritz S, Berger T, Pop V, Spek V, Cuijpers P (2017). Efficacy of self-guided internet-based cognitive behavioral therapy in the treatment of depressive symptoms: a meta-analysis of individual participant data. JAMA Psychiatry.

[31] Khan K, Hall CL, Davies EB, Hollis C, Glazebrook C (2019). The effectiveness of web-based interventions delivered to children and young people with neurodevelopmental disorders: systematic review and meta-analysis. J Med Internet Res.

[32] Rogers MA, Lemmen K, Kramer R, Mann J, Chopra V (2017). Internet-delivered health interventions that work: systematic review of meta-analyses and evaluation of website availability. J Med Internet Res.

[33] Scholten MR, Kelders SM, Van Gemert-Pijnen JE (2017). Self-guided web-based interventions: scoping review on user needs and the potential of embodied conversational agents to address them. J Med Internet Res.

[34] Andersson G, Cuijpers P, Carlbring P, Riper H, Hedman E (2014). Guided Internet-based vs. face-to-face cognitive behavior therapy for psychiatric and somatic disorders: a systematic review and meta-analysis. World Psychiatry.

[35] Carlbring P, Andersson G, Cuijpers P, Riper H, Hedman-Lagerlöf E (2018). Internet-based vs. face-to-face cognitive behavior therapy for psychiatric and somatic disorders: an updated systematic review and meta-analysis. Cogn Behav Ther.

[36] Talbot F (2012). Client contact in self-help therapy for anxiety and depression: necessary but can take a variety of forms beside therapist contact. Behav Change.

[37] Goslar M, Leibetseder M, Muench HM, Hofmann SG, Laireiter A (2017). Efficacy of face-to-face versus self-guided treatments for disordered gambling: a meta-analysis. J Behav Addict.

[38] Dziobek I, Lipinski S (2021). Participatory research in clinical psychology and psychiatry in Germany – achievements, implementation, and a look to the future. Psychiatr Prax.

[39] Levitan B, Getz K, Eisenstein EL, Goldberg M, Harker M, Hesterlee S, Patrick-Lake B, Roberts JN, DiMasi J (2018). Assessing the financial value of patient engagement: a quantitative approach from CTTI's patient groups and clinical trials project. Ther Innov Regul Sci.

[40] von Peter S (2017). Participatory and collaborative strategies in psychiatric research. Psychiatr Prax.

[41] Chen Y, Goh K, Razak M (2012). Development of a web-based tailored intervention for excessive gaming. Proceedings of the 2012 World Congress Conference on Engineering and Computer Science.

[42] Hanke S, Brecht L, Petersen K, Barth GM, Renner T, Batra A, Brandhorst I (2022). Preparation of an online training programme for the parents of adolescents and young adults with internet use disorders. Kindh Entwickl.

[43] Bernstein K, Zarski A, Pekarek E, Schaub MP, Berking M, Baumeister H, Ebert DD (2023). Case report for an internet- and mobile-based intervention for internet use disorder. Front Psychiatry.

[44] Chau C, Tsui YY, Cheng C (2019). Gamification for internet gaming disorder prevention: evaluation of a wise IT-use (WIT) program for Hong Kong primary students. Front Psychol.

[45] Dominguez-Rodriguez A, De La Rosa-Gómez A (2022). A perspective on how user-centered design could improve the impact of self-applied psychological interventions in low- or middle-income countries in Latin America. Front Digit Health.

[46] Larsson Lund M, Månsson Lexell E, Nyman A (2022). Optimising the development of sustainable internet-based occupational therapy interventions: important key actions and perspectives to consider. Scand J Occup Ther.

[47] Kuckartz U, Rädiker S (2022). Qualitative Content Analysis: Methods, Practice and Software.

[48] Tong A, Sainsbury P, Craig J (2007). Consolidated criteria for reporting qualitative research (COREQ): a 32-item checklist for interviews and focus groups. Int J Qual Health Care.

[49] Helfferich C (2011). The Quality of Qualitative Data. A Manual for Conducting Qualitative Interviews.

[50] Mayring P (2022). Qualitative Inhaltsanalyse: Grundlagen und Techniken.

[51] Clarke V, Braun V, Hayfield N, Smith JA (2015). Thematic analysis. Qualitative Psychology: A Practical Guide to Research Methods. 3rd edition.

[52] Sabnis SV, Wolgemuth JR (2023). Validity practices in qualitative research in school psychology. Sch Psychol Int.

[53] ISO 9241-210:2019: ergonomics of human-system interaction: part 210: human-centred design for interactive systems. International Organization for Standardization (ISO).

[54] Bosworth T, Williams A, Wilson G, Flowers L (2023). Development and design needs of mobile health (mHealth) apps for adolescents. Ann Fam Med.

[55] Brooke J (1995). SUS: a quick and dirty usability scale. ResearchGate.

[56] Rollo S, Venuleo C, Ferrante L, De Luca Picione R (2023). What adolescents have to say about problematic internet use: a qualitative study based on focus groups. Int J Environ Res Public Health.

[57] Wartberg L, Lindenberg K (2020). Predictors of spontaneous remission of problematic internet use in adolescence: a one-year follow-up study. Int J Environ Res Public Health.

[58] Lin PY, Lin HC, Lin PC, Yen JY, Ko CH (2020). The association between emotional regulation and internet gaming disorder. Psychiatry Res.

[59] Yen JY, Yeh YC, Wang PW, Liu TL, Chen YY, Ko CH (2017). Emotional regulation in young adults with internet gaming disorder. Int J Environ Res Public Health.

[60] Villani D, Carissoli C, Triberti S, Marchetti A, Gilli G, Riva G (2018). Videogames for emotion regulation: a systematic review. Games Health J.

[61] Gurdal S, Kapetanovic S, Einarsson I, Boson K, Claesdotter-Knutsson E (2023). Adolescents' perceptions of a relapse prevention treatment for problematic gaming-a qualitative study. Healthcare (Basel).

[62] Surmann M, Gruchalla LV, Falke S, Maisch B, Uhlmann C, Bock E, Arolt V, Lencer R (2017). The importance of strengthening competence and control beliefs in patients with psychosis to reduce treatment hindering self-stigmatization. Psychiatry Res.

[63] Matthews S, Dwyer R, Snoek A (2017). Stigma and self-stigma in addiction. J Bioeth Inq.

[64] Sugaya N, Shirasaka T, Takahashi K, Kanda H (2019). Bio-psychosocial factors of children and adolescents with internet gaming disorder: a systematic review. Biopsychosoc Med.

[65] Bendotti H, Lawler S, Chan GC, Gartner C, Ireland D, Marshall HM (2023). Conversational artificial intelligence interventions to support smoking cessation: a systematic review and meta-analysis. Digit Health.

[66] Holter MT, Ness O, Johansen AB, Brendryen H (2020). Making come-alive and keeping un-alive: how people relate to self-guided web-based health interventions. Qual Health Res.

[67] Geisler BL (2023). Lebensbewältigung im Spiegel der Internetnutzung: Eine qualitative Biografiestudie zur problematischen Internetnutzung bei Frauen.

[68] Kim HS, Son G, Roh EB, Ahn WY, Kim J, Shin S, Chey J, Choi K (2022). Prevalence of gaming disorder: a meta-analysis. Addict Behav.

[69] (2020). Youth-centred digital health interventions: a framework for planning, developing and implementing solutions with and for young people. World Health Organization.

