# Supplementary material for: Ambivalent User Needs as a Challenge and Chance for the Design of a Web-Based Intervention for Gaming Disorder: Qualitative Interview Study With Adolescents and Young Adults
Source: JMIR Form Res. 2025 May 26;9:e63258. doi: 10.2196/63258 (PMC12149767; doi:10.2196/63258)
Supplement: Multimedia Appendix 2 [file formative_v9i1e63258_app2.docx]

Appendix

**Code Tree**

| **Coding categories**  (superordinate categories and main categories) | **Description** |
| --- | --- |
| 1. Experiences with gaming disorder | |
| 1.1 Needs satisfied by games  1.2 Negative consequences of gaming 1.3 Risk contexts | This category was used to code data related to problematic experiences with gaming. |
| 2. Experiences with successful strategies in coping with GD symptoms | |
| 2.1 Using external stabilizers  2.2 Not expecting too much of oneself/avoiding extremes  2.3 Self-reflection/knowing oneself  2.4 Reducing gaming/creating distance from gaming  2.5 Having alternative activities ready as a substitute  2.6 Taking personal responsibility | This category was used to code data related to successful experiences with strategies outside the Internet for coping with GD. |
| 3. Barriers to using strategies in coping with GD symptoms | |
| 3.1 Not enough pressure or urgency  3.2 Too much pressure or urgency  3.3 Too narrow framing of the problem | This category was used to code data related to barriers participants experienced when using strategies outside the Internet to cope with GD. |
| 4. Attitudes and beliefs towards gaming | |
| 4.1 Lack of agency  4.2 Having agency  4.3 Dopamine hypothesis or seeking the ‘high’  4.4 Symptom is not cause  4.5 Gaming as a ‘better alternative’ to the real world  4.6 Gaming as an educational and cultural resource | This category was used to code data related to beliefs and attitudes about gaming and GD. |
| 5. Motivations to accept help for changing the gaming behavior | |
| 5.1 Opportunity to connect with others  5.2 Autonomy and making own decision  5.3 Positive, solution-oriented attitude  5.4 Suffering | This category was used to code data related to participants’ motivation to accept help regarding GD in the past. |
| 6. Expectations of an online training in general | |
| 6.1 High quality design  6.2 Comprehensibility  6.3 Providing a challenge  6.4 Enabling autonomy | This category was used to code data related to general user needs regarding web-based help, interpreted from positive and negative experiences. |
| 7. Expectations of a self-guided WBI for GD | |
| 7.1 Connection to the life environment of the individual  7.2 Enabling self-reflection  7.3 Integration of a personal counseling service  7.4 Attractive design  7.5 Enabling the creation / tracking of a plan for change | This category was used to code data related to explicitly formulated expectations of a potential self-guided WBI for GD. |
| 7.6 Online training as a bridge to the offline local treatment service  7.7 Duration and frequency of use  7.8 Flexible/low-threshold access  7.9 Optimal promotion |  |
| 8. Expectations about barriers to using online training for GD | |
| 8.1 Negative framing 8.2 Lack of differentiation 8.3 Not reflecting the reality of the target audience's lives 8.4 Use of game elements 8.5 Focus on digital help only | This category was used to code data related to hypothetical barriers that participants expected to encounter if they actually used a self-guided WBI for GD. |

This is a Multimedia Appendix to a full manuscript published in the JMIR Formative Research.
