# Supplementary material for: Ambivalent User Needs as a Challenge and Chance for the Design of a Web-Based Intervention for Gaming Disorder: Qualitative Interview Study With Adolescents and Young Adults
Source: JMIR Form Res. 2025 May 26;9:e63258. doi: 10.2196/63258 (PMC12149767; doi:10.2196/63258)
Supplement: Multimedia Appendix 3 [file formative_v9i1e63258_app3.docx]

Appendix

**Theme matrix (example): Expectations of a self-guided WBI for GD**  The white boxes contain the subcategories.

| Superordinate category | **Expectations of a self-guided WBI for GD** | **Participant 1**  The online training should… | **Participant 2**  The online training should… | **Participant 3**  The online training should… | **Participant 4**  The online training should… | **Participant 5**  The online training should… | **Participant 6**  The online training should… |
| --- | --- | --- | --- | --- | --- | --- | --- |
| Main  categories | *Connection to the life environment of the individual* | - not trigger a gaming disorder. > It should be usable on a separate device that is not connected to the Internet. - The participant is ambivalent about sharing experiences with other people with gaming disorder in the online training. | - not trigger a gaming disorder. > Therefore ambivalence about whether reward systems in online training serve as motivation or as a trigger for gaming disorder. - be an advantage because of its proximity to the online game. -> It can meet people with gaming disorder where they are (on the Internet). - mediate that there are many others who are also affected by a gaming disorder. | - not trigger a gaming disorder. > Therefore ambivalence about whether reward systems in online training serve as motivation or as a trigger for gaming disorder. | - provide authentic and "real" content. | - provide a self-help community to interact with others affected by gaming disorder. | - provide authentic and "real" content. - be based on experience, not on research. - get into the mindset of people affected by a gaming disorder. - provide regular process support, but with "real talk". - avoid the term "addiction" in the title of the course. - offer content from the perspective of someone with a gaming disorder and has been through it. - Include regular people as key players. |
|  | *Enabling  self-reflection* | - enable a first step in problem reflection. - provide an objective, non-judgmental assessment of the negative consequences of gaming. | - enable a first step in problem reflection. | - provide an objective, non-judgmental assessment of the negative consequences of gaming. | - enable a first step in problem reflection. | - allow entry of hobbies, daily structure, and play time at the beginning. - offer orientation questions at the beginning. - offer self-reflection questions about gaming behavior. - allow tracking of one's gaming behavior over a period of time. | --- |
|  | *Integration of a personal counseling service* | -- | --- | --- | --- | - enable regular counselor contact as training support - provide the ability to choose the right counselor - allow the counselor to be available via chat for urgent questions - offer a combination of counseling and self-directed tasks - offer counseling for parents as well as three-way meetings - allow sharing of status messages (pictures, videos) with parents/counselors | - offer contact with counselor directly via Zoom rather than by email - provide regular process support, but with "real talk". |
|  | *Attractive  design* | - be designed in an attractive way to make the user want to use it. | - be designed in an attractive way to make the user want to use it. - include game elements > to impart knowledge in a playful way. - be used as effortlessly as possible. | - include game elements > to impart knowledge in a playful way. | - focus on success rather than problems > help get life back on track. - offer an attractive, varied design > video-based rather than text-based content. - give impulses that make you think. | - offer an attractive, varied design > should not always be the same. - focus on success rather than problems > motivating reward elements (coins, avatars). - include game elements > encouraging tasks and games; games that are adapted to the level of competence; games for less strenuous learning; tasks that are relevant and worthwhile. - include explanatory elements (ideally via video) on how the training works. | - offer an attractive, varied design > video-based rather than text-based content. - focus on success rather than problems > motivational videos from influencers instead of focusing on reducing gaming; motivating with the benefits of what you can achieve in life without a gaming disorder. - motivate with reflection of successes |
|  | *Enabling the creation/ tracking of a plan for change* | - enable the solution-oriented development of a vision/goal. - provide change strategies. | --- | --- | - provide change strategies. | - allow for discussion of gaming behavior and development of a plan for change. - allow the implementation of the plan for change to be tracked. - provide change strategies. | --- |
|  | *Online training as a bridge to the offline local treatment service* | - serve as a bridge to the analogous on-site counseling offer. | - serve as a bridge to the analogous on-site counseling offer. | --- | --- | --- | --- |
|  | *Duration and frequency of use* | - be designed for a usage time of 30 min. | - be designed for a usage time of 15 to 30 min. | - be designed for a usage time of 15 min. | - be designed based on short videos with a usage time of 3 to 5 min. | - be designed for a usage time for 15 min. per day. - Include counseling sessions lasting 30 to 45 min. - be designed for usage several times per week. | - be usable once for 30 min. |
|  | *Flexible/ low-threshold  access* | -- | - be easy to start without registration - be browser-based | --- | - be spontaneously usable in critical situations. - be usable across devices. | - be usable across devices. - include educational content for both parents and children. - be usable especially on PC and mobile phone. | - be video-based. |
|  | *Optimal  promotion* | --- | - be easy to find. - be promoted via video-based advertising in social networks. | - be promoted via video-based advertising in social networks > by offering a  YouTube channel; by advertising with the help of an influencer. | - be easy to find. - be promoted via video-based advertising in social networks. | - be promoted via video-based advertising in social networks. > Youtube and TikTok | - be promoted via video-based advertising in social networks. - be promoted via video-based advertising in social networks. > A therapist in a recorded video as a promotional tool. - be promoted via advertising that shows successes. - be promoted via advertising that scares with negative consequences. |

This is a Multimedia Appendix to a full manuscript published in the JMIR Formative Research.
